# The Smartphone Addiction Scale: Development and Validation of a Short Version for Adolescents

**DOI:** 10.1371/journal.pone.0083558

**Published:** 2013-12-31

**Authors:** Min Kwon, Dai-Jin Kim, Hyun Cho, Soo Yang

**Affiliations:** 1 Addiction Research Institute, Department of Psychiatry, Seoul St. Mary's Hospital, The Catholic University of Korea, Seoul, South Korea; 2 Department of Psychiatry, Seoul St. Mary's Hospital, College of Medicine, The Catholic University of Korea, Seoul, South Korea; 3 College of Nursing, The Catholic University of Korea, Seoul, South Korea; Mayo Clinic College of Medicine, United States of America

## Abstract

**Objective:**

This study was designed to investigate the revised and short version of the smartphone addiction scale and the proof of its validity in adolescents. In addition, it suggested cutting off the values by gender in order to determine smartphone addiction and elaborate the characteristics of smartphone usage in adolescents.

**Method:**

A set of questionnaires were provided to a total of 540 selected participants from April to May of 2013. The participants consisted of 343 boys and 197 girls, and their average age was 14.5 years old. The content validity was performed on a selection of shortened items, while an internal-consistency test was conducted for the verification of its reliability. The concurrent validity was confirmed using SAS, SAPS and KS-scale. Receiver operating characteristics analysis was conducted to suggest cut-off.

**Results:**

The 10 final questions were selected using content validity. The internal consistency and concurrent validity of SAS were verified with a Cronbach's alpha of 0.911. The SAS-SV was significantly correlated with the SAS, SAPS and KS-scale. The SAS-SV scores of gender (p<.001) and self-evaluation of smartphone addiction (p<.001) showed significant difference. The ROC analysis results showed an area under a curve (AUC) value of 0.963(0.888–1.000), a cut-off value of 31, sensitivity value of 0.867 and specificity value of 0.893 in boys while an AUC value of 0.947(0.887–1.000), a cut-off value of 33, sensitivity value of 0.875, and a specificity value of 0.886 in girls.

**Conclusions:**

The SAS-SV showed good reliability and validity for the assessment of smartphone addiction. The smartphone addiction scale short version, which was developed and validated in this study, could be used efficiently for the evaluation of smartphone addiction in community and research areas.

## Introduction

Nowadays, addiction not only refers to drug or substance abuse, but it also refers to gambling, internet, games, or even smartphones. These also fall under the category of behavioral addiction [1]. The conventional diagnostic system strictly regards only symptoms caused by repetitive and excessive substance use as addiction. However, not only average people but even clinicians use the term ‘addiction’ when a person is obsessed with a certain activity that results in disturbance of his/her daily activities and shows a pattern similar to substance dependence. The classic example of this activity is gambling. In the conventional diagnostic system, gambling is classified as an impulse control disorder; but after continuous studies on its similarity with substance addiction, it was considered addiction in the DSM-5 based on the results of studies on its neurobiological and psychopathological evidence. Recently, it has been reported that internet based activities such as gaming, chatting and pornography have been showing similar levels of addiction as those of drug and substance abuse. Moreover, as the internet become more accessible through smartphone, the addiction pattern associated with smartphone has been shown more routinely and the concerns relating the phenomenon have increased.

This new type of addiction has been caused by fast-developing media including internet and smartphones in advanced IT industries. It has caught the attention of countries all over the world. Owing to South Korea's advanced IT development, quick access to the internet and fast distribution of smartphones resulted in a serious behavioral addiction, mostly noticeable in a vulnerable class of people including adolescents. According to the survey of smartphone addiction completed by the National Information Society Agency in 2012, the percentage of smartphone addiction was 8.4%, which was found to be higher than the internet addiction of 7.7% [2]. The higher figure in teenagers to individuals in their twenties than in those in their thirties to forties implies that this problem may worsen in the future.

The hardware and software of smartphones are dramatically improving and various applications are being developed and available to suit our lifestyle. This change is beyond our imagination. A smartphone functions not only as a mobile phone but also as a computer, mp3 or video player. You can easily gain access to any information that you want at any given time or place. It also has many other advantages including, but not limited to, entertainment. As a result, many people in this modern society are extremely interested in acquiring a smartphone.

However, considering its mobile and internet capabilities, a smartphone also has the possibility of becoming a prevalent social problem as it signifies the characteristics of addiction such as tolerance, withdrawal, difficulty of performing daily activities, or impulse control disorders as confirmed in previous studies. Kuss & Griffiths have mentioned the possibility of a social networking sites (SNS) addiction through their online social networking study and Park & Lee also reported that smartphone use could be attributed to loneliness, depression and self-esteem based on their smartphone use and psychological well-being study [3]–[4]. China reported the psychological risk factors of addiction to social networking sites by investigating outcome expectancies, impulsivity and internet self-efficacy in Chinese smartphone users [5]. Other studies conducted by South Korea's university students have also proven the relationship of smartphone addiction to mental health, campus life, personal relations, self-control and life stress [6]–[7]. Korea reported that adolescents may be candidates for a higher risk of exhibiting these problems with the use of smartphones than the adults because the adolescents use smartphones as an alternative way to access the internet as shown in their new patterns through the media addiction study [8].

One of the reasons for this unexpected popularity of smartphones is that it makes people's lives more convenient. However, this may also pose many risks for such dependence over a gadget. This ‘smartphone addiction’ recently has become an important issue in our society. According to the study related to the development of smartphone addiction scale, smartphones also caused symptoms of addiction similar to the effects of the internet including craving, withdrawal, tolerance, daily-life disturbance, and preference of cyberspace-oriented relationship, which were confirmed through the diagnosis [9].

There are few international journals published related to smartphone addiction. In the Korean journals, the rating scales of studies that were done simply used modified terminologies from previous researches rather than those based from understanding the concept of smartphone addiction. The cut-off value was based on simple evaluation using statistical methods. The figure is vague; therefore, the use of those scales is limited. In order to overcome the limitation of the previous scales, Kwon et al. have developed and validated the SAS(Smartphone Addiction Scale), which consisted of 33 questions and 6 points, to evaluate the smartphone addiction using self-reporting. The following six factors were considered in the questionnaire, I would say, daily-life disturbance, positive anticipation, withdrawal, cyberspace-oriented relationship, overuse, and tolerance. However, this scale conducted in university students and adults showed limited results due to the study participants and their ages. Furthermore, the ratio between male and female was imbalanced, so it was difficult to compare the difference between genders. Lastly, the cut-off value was not suggested to evaluate the addiction degree.

This study was designed to develop a short-version scale in order to evaluate the degree of addiction in teenagers as well as to suggest a cut-off value by gender using the previous scale and major concept of addiction for evaluation reference. In addition, characteristics of smartphone usage in participants are also investigated. The short-version scale developed from this study is expected to evaluate smartphone addiction in a simple and easy way, which will be less expensive and time consuming. The cut-off value can also be used in clinical and practical fields of society as well as other studies. The authors are expecting that this scale will be used efficiently for the screening of smartphone addiction in community areas and further studies, and for the evaluation of the treatment progress.

## Materials and Methods

### Participants

The participants of this study were 599 students in their 2^nd^ year of junior high school from two schools in the Kangwon province of South Korea. However, 59 of those students did not use smartphones or were unable to complete the set of questionnaires, and they were excluded from the analysis. Statistical analysis on the EM algorithm was used for missing data. As a result, the only valid analysis results were taken from 540 students, which were 343 boys and 197 girls. Their average age was 14.5.

This study was conducted after the written consent of the anonymous volunteer participants was obtained. The ethical standards in the 1964 Declaration of Helsinki were followed. Each participant and the caretakers or guardians on the adolescent participants provided a written informed consent after receiving a full explanation of the study's purpose and procedure that has been approved by the Institutional Review Board of Seoul St. Mary's Hospital. This study was approved by the ethics committee of Catholic Medical Center Office of Human Research Protection Program (reference KC13ONSI0080).

### Measurement

1. **SAS.** Smartphone addiction scale (SAS) is a scale for smartphone addiction that consisted of 6 factors and 33 items with a six-point Likert scale (1: “strongly disagree” and 6: “strongly agree”) based on self-reporting. The six factors were daily-life disturbance, positive anticipation, withdrawal, cyberspace-oriented relationship, overuse, and tolerance. During its development stages, the internal-consistency test result (Cronbach's alpha) was 0.967. In this study, the internal-consistency test result (Cronbach's alpha) of SAS was 0.966.
2. **SAPS.** Smartphone Addiction Proneness Scale (SAPS) was added to verify the concurrent validity of SAS-SV. Based on previously published internet addiction scales and celluar phone addiction scale, SAPS was developed [10]. The SAPS consisted of 15 items scored with a four-point Likert scale (1: “not at all” and 4: “always”). The reliability test of the scale yielded a Cronbach's alpha of .880. In this study, the internal-consistency test result (Cronbach's alpha) of SAPS was 0.861.
3. **KS-scale.** The Korean self-reporting internet addiction scale short-form scale (KS-scale) was added to verify the concurrent validity of SAS-SV. KS-scale was developed in collaboration with the Korea Agency for Digital Opportunities and Seoul National University [11]. The KS-scale consisted of 20 items scored with a four-point Likert scale (1: “not at all” and 4: “always”). According to the scores of the KS-scale total and subscales, students were classified into high risk for internet addiction, potential risk for internet addiction, and general user groups. As a result of the reliability analysis of the KS-scale, the Cronbach's alpha for middle school student scores was 0.909. In this study, the internal-consistency test result (Cronbach's alpha) of the KS-scale was 0.836.

### Statistical Analysis

1. **Content Validity Index of SAS.** In order to shorten the previous version of SAS consisting of 33 questions, a content validity using CVI recommended by Polit & Beck was conducted by 7 experts [12]. The objective of this scale was explained to the experts, namely, 3 psychiatrists (M.D.), 2 nurses with doctorate degree, and psychologists with doctorate degree. They were asked to review the questions after fully comprehending the terminology. Afterwards, they were asked to select the questions that have to be included in the scale to evaluate addiction based on their specialty to determine the final set of questions. To evaluate content validity, the experts rated the relevance of each item using the 4-point ordinal rating scale (1: “an irrelevant item”, 2: “unable to assess the relevance without item revision”. 3: “relevant but needs minor alteration”, and 4: “an extremely relevant item”). The actual CVI was a proportion of the items that received a rating of 3 or 4 by the experts.
2. **Socio-demographic characteristics and SAS-SV scores.** Descriptive statistics and frequency analysis were used to analyze socio-demographic characteristics of the participants including information related to self-assessment of smartphone addiction. The difference on the SAS-SV scores of each item was analyzed using t-test and ANOVA (mean comparison), while the Scheffé test was performed as a post-hoc test.
3. **Internal Consistency Reliability for SAS-SV.** In order to check the reliability of each item, Cronbach's alpha correlation coefficient was confirmed for each question and scale of 10 questions.
4. **Concurrent validity of SAS-SV.** Pearson's correlation analysis of SAS, SAPS and KS-scale was measured in order to determine the degree of smartphone addiction. Moreover, in order to check the characteristics of gender, an additional analysis was conducted in each gender group.
5. **ROC curve of SAS-SV.** Receiver operating characteristics (ROC) analysis was conducted to examine the diagnostic ability of the SAS-SV for predicting smartphone addiction. The area under the curve (AUC) of the ROC is a measurement of the diagnostic ability of the SAS-SV score in order to correctly classify a specified outcome of smartphone addiction diagnosis through consultation with clinical psychologists. The clinical psychologists selected 90 boys and 60 girls among all the participants using simple random sampling from a computer program. The addiction group was determined after their consultation based on tolerance, withdrawal and daily-life disturbance, which were among the general symptoms of addiction.

A value of 1.0 is considered as a perfect test, whereas a value of .5 or smaller is considered similar to a random guess or flipping a coin based on its inconsistency. The guidelines for interpreting the AUC values were as follows: AUC of 0.7–0.8 is acceptable, AUC of 0.8–0.9 is excellent, and AUC of 0.9 or greater is outstanding [13]. The value corresponding to the nearest point of the ROC curve to the top left-hand corner was chosen as the optimal cut-off for predicting smartphone addiction wherein it maximizes both sensitivity and specificity.

## Results

### Question selection for short-version scale

Based on the validity reviewed by experts, 10 out of 33 questions have been selected. The questions were selected by at least 6 experts and showed more than CVI of 0.78. The mean I-CVI was 0.943 and the S-CVI/UA was 0.60. The final questions were selected using SAS-SV as shown in table 1.

**Table 1:** Content Validity Index of SAS.

Items		Number in Experts' Agreement	Item CVI
1	Missing planned work due to smartphone use	7	1.000
2	Having a hard time concentrating in class, while doing assignments, or while working due to smartphone use	7	1.000
3	Experiencing lightheadedness or blurred vision due to excessive smartphone use	5	.714
4	Feeling pain in the wrists or at the back of the neck while using a smartphone	6	.857
5	Feeling tired and lacking adequate sleep due to excessive smartphone use	4	.571
6	Feeling calm or cozy while using a smartphone	1	.143
7	Feeling pleasant or excited while using a smartphone	4	.571
8	Feeling confident while using a smartphone	3	.429
9	Being able to get rid of stress with a smartphone	3	.429
10	There is nothing more fun to do than using my smartphone.	3	.429
11	My life would be empty without my smartphone.	4	.571
12	Feeling most liberal while using a smartphone	0	.000
13	Using a smartphone is the most fun thing to do.	5	.714
14	Won't be able to stand not having a smartphone	7	1.000
15	Feeling impatient and fretful when I am not holding my smartphone	7	1.000
16	Having my smartphone in my mind even when I am not using it	7	1.000
17	I will never give up using my smartphone even when my daily life is already greatly affected by it	6	.857
18	Getting irritated when bothered while using my smartphone	5	.714
19	Bringing my smartphone to the toilet even when I am in a hurry to get there	4	.571
20	Feeling great meeting more people via smartphone use	2	.286
21	Feeling that my relationships with my smartphone buddies are more intimate than my relationships with my real-life friends	5	.714
22	Not being able to use my smartphone would be as painful as losing a friend.	4	.571
23	Feeling that my smartphone buddies understand me better than my real-life friends	3	.429
24	Constantly checking my smartphone so as not to miss conversations between other people on Twitter or Facebook	6	.857
25	Checking SNS (Social Networking Service) sites like Twitter or Facebook right after waking up	5	.714
26	Preferring talking with my smartphone buddies to hanging out with my real-life friends or with the other members of my family	3	.429
27	Preferring searching from my smartphone to asking other people	1	.143
28	My fully charged battery does not last for one whole day.	2	.286
29	Using my smartphone longer than I had intended	7	1.000
30	Feeling the urge to use my smartphone again right after I stopped using it	4	.571
31	Having tried time and again to shorten my smartphone use time, but failing all the time	5	.714
32	Always thinking that I should shorten my smartphone use time	4	.571
33	The people around me tell me that I use my smartphone too much	6	.857
		Mean I-CVI	.943
		S-CVI/UA	.600

I-CVI, item-level content validity index.

S-CVI/UA, scale-level content validity index, universal agreement calculation method.

### Socio-demographic characteristics and SAS-SV scores of the participants

Out of 540 participants, 343 (63.5%) were boys and 197 (36.5%) were girls. Their SAS-SV scores were 23.75 and 27.89, respectively, which showed significant difference (p<0.001). In a socio-economic state, the medium was 191 (53.2%). As for family environments, 69 (12.8%) students have single parents while 23 (4.3%) students do not have parents. However, those characteristics did not show significant difference on the SAS-SV score. The number of students who drink was 21 (4.1%) and the number of students who smoke was 22 (4.2%). Their SAS-SV scores were 27.52 and 27.94, respectively. They were also not statistically significant, but they were higher than those who did not drink or smoke (see table 2).

**Table 2:** Socio-demographic characteristics and SAS-SV scores.

				(N = 540)
Variables		N (%)	SAS-SV	*p*
		Mean±SD	Mean±SD	
Age		14.46±0.54		
Sex	Boy	343(63.5)	23.75±10.26	<.001
	Girl	197(36.5)	27.89±11.17	
Socio-economic state	Low	86(24.0)	25.08±10.65	.828
	Medium	191(53.2)	25.90±11.10	
	High	82(22.8)	25.94±11.12	
Family	Parents	442(81.9)	25.53±11.03	.173
	Single-parent	69(12.8)	23.07±9.62	
	No-Parent	23(4.3)	26.70±9.61	
Alcohol	Yes	21(4.1)	27.52±9.98	.295
	No	496(95.9)	25.03±10.69	
Smoking	Yes	22(4.2)	27.94±10.91	.210
	No	496(95.8)	25.03±10.61	
Time of smartphone use	Weekday	3.03±3.09		
	Weekend	4.07±4.08		
Purpose of smartphone use	Messenger and SNS	300(55.6)	26.29±11.07	.077
	Entertainment	114(21.1)	23.42±10.03	
	Web surfing	43(8.0)	24.79±9.88	
	Etc.	83(15.4)	24.29±10.86	
Self evaluation of smartphone addiction	Non-addiction	312(57.8)	21.82±8.34^a^	<.001
	Addiction	134(24.8)	34.02±10.34^ab^	
	Don't know	94(17.4)	24.17±11.68^b^	

^a, b^ : Scheffé test (the means with the same letter were significantly different).

As for the usage of smartphones, the average hours were 3.03 during weekdays and 4.07 during weekends. Based on the conducted research on 300 (55.6%) students, which was over 50%, the use of the messenger or SNS was the major purpose of a smartphone, followed by 114 (21.1%) students who used them for entertainment purposes such as listening to music, watching movies, or playing games. In the self-assessment of smartphone addiction, 134 (24.8%) students considered themselves as addicted to smartphones, 312 (57.8%) students considered themselves as not addicted to smartphones, and 94 (17.4%) students were unsure. The SAS-SV scores among them had statistically significant difference (p<0.001), which showed 34.02 in people who considered themselves addicted to smartphones (see table 2).

### Internal Consistency Reliability of the SAS-SV

The SAS-SV scores and standard deviations of each item were shown in table 3. The total mean was 25.26. A Cronbach's alpha correlation coefficient of 0.91 was obtained for the SAS-SV. The corrected item total correlation coefficients ranged from 0.50 to 0.80 and were well within the acceptable range as prescribed by Nunnally & Bernstein [14]. The deletion of any individual item would have decreased the internal consistency reliability as all of them showed values of more than 0.90.

**Table 3:** Reliability for SAS-SV.

					(N = 540)
Items		Item	Standard	Corrected	Alpha if Item
		Mean	Deviation	Item/Total	Deleted
1	Missing planned work due to smartphone use	2.78	1.53	.66	.903
2	Having a hard time concentrating in class, while doing assignments, or while working due to smartphone use	2.56	1.48	.72	.900
3	Feeling pain in the wrists or at the back of the neck while using a smartphone	2.72	1.51	.66	.903
4	Won't be able to stand not having a smartphone	2.40	1.44	.68	.902
5	Feeling impatient and fretful when I am not holding my smartphone	2.12	1.32	.72	.900
6	Having my smartphone in my mind even when I am not using it	2.28	1.32	.74	.899
7	I will never give up using my smartphone even when my daily life is already greatly affected by it.	2.19	1.26	.69	.902
8	Constantly checking my smartphone so as not to miss conversations between other people on Twitter or Facebook	2.49	1.47	.57	.909
9	Using my smartphone longer than I had intended	3.02	1.55	.68	.902
10	The people around me tell me that I use my smartphone too much.	2.70	1.55	.67	.903

Overall alpha = .911; Scale Mean = 25.26; SD = 10.78.

### Concurrent Validity of the SAS-SV: Correlations between SAS, SAPS and KS-scale

In order to determine the concurrent validity of the SAS-SV, the correlations between SAS, SAPS, KS-scale were investigated. The results were shown in Table 4. Each couple was significantly related. The correlation between SAS and KS-scale, which evaluated internet addiction, showed 0.611 in boys and 0.375 in girls. This has proven a difference by gender group.

**Table 4:** Concurrent validity of SAS-SV.

			(N = 540)
	SAS	SAPS	KS-scale(IAD)
SAS-SV	.958**	.762**	.421**
SAS-SV(Boy)	.958**	.694**	.611**
SAS-SV(Girl)	.959**	.855**	.375**

SAPS: Kim's Smartphone Addiction Proneness Scale.

*p*<.001.

### The Diagnostic Ability of the SAS-SV

**Figure 1:**
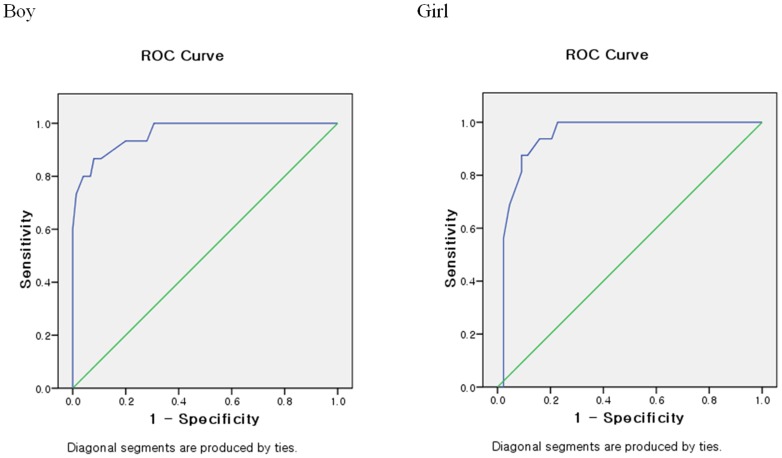
Receiver operating characteristics curve for the Smartphone Addiction Scale – Short Version score to predict smartphone addiction compared with gender.

As shown in Table 5, ROC analysis was performed in order to predict smartphone addiction. A random selection of 90 boys and 60 girls was done and they underwent consultation with clinical psychologists. The symptoms of addiction, tolerance and withdrawal, were exhibited by 15 (16.6%) boys and 16 (26.6%) girls. They were considered as addicted to smartphones. The cut-off value of SAS-SV was determined based on the consultation results with the clinical psychologists. Figure 1 showed the ROC curve by gender. In boys, the AUC value was 0.963 (0.888–1.000), the cut-off value was 31, sensitivity value was 0.867, and specificity value was 0.893. As for the girls, the AUC value was 0.947(0.887–1.000), the cut-off value was 33, sensitivity value was 0.875, and specificity value was 0.886. Based on the cut-off values, this scale was considered as an appropriate tool for evaluating smartphone addiction.

**Table 5:** Summary of ROC Analysis Predicting Smartphone Addiction.

	AUC	95% CI	Cut-off	Sensitivity	Specificity	PPV	NPV
SAS-SV(Boy)	0.963	0.888–1.000	31	0.867	0.893	0.619	0.971
SAS-SV(Girl)	0.947	0.887–1.000	33	0.875	0.886	0.737	0.951

AUC, area under the curve; CI, confidence interval; PPV, positive predictive value; NPV, negative predictive value.

## Discussion

Kim et al. reported that adolescents have the tendency to concentrate while using media and can develop more habitual usage problems than adults when they are introduced to a new type of media [8]. In other words, adolescents tend to proactively accept new media and substitute the previous one. Based on comparisons with adults, teenagers are more vulnerable to smartphone addiction. As a result, preventive measures should be taken into consideration and the adolescents, who were predicted to develop smartphone addiction, should be identified. In addition, the importance of cut-off values, which were suggested through the development and validation of smartphone addiction scale, had been highlighted.

The general use of smartphones showed an SAS-SV score of 26, which was the highest in the usage of messenger or SNS applications compared with other forms of use. The detailed research showed that the messenger or SNS included Facebook, Twitter, and popular applications in South Korea such as Kakao Story and Kakao game, which were both connected to the messenger. The use of these applications was considered to reflect the social characteristics of SNS as people were able to play games and interact with their friends and acquaintances. As for the self-assessment of smartphone addiction, the group who assessed themselves as addicted to smartphones showed 34 points in the SAS-SV score, while the group who assessed themselves as not addicted to smartphone was 22. This result tells us that the high SAS-SV score reflects the self-awareness about the seriousness of smartphone addiction.

In our concurrent validity test, the previous SAS and SAPS showed over 0.70, which was a high correlation. However, the KS-scale showed moderate correlation. This result showed that the propensity of smartphone addiction and internet addiction was not completely identical, but rather they had moderate correlation [15].

As for the use of smartphones, the previous studies on mobile phones showed different usage patterns or propensities of addiction by gender. Billieux et al. reported the difference on the psychological processes with regards to the use of a mobile phone as motivational aspects by gender, especially in the female participants who showed focused disposition of social interaction than the male participants, and suggested that further study was necessary on the new possible aspects by gender [16]. In addition, Takao et al. reported that the problem was more serious in the female participants in the study of mobile phone usage problems [17].

Other studies on smartphone addiction, which were conducted in South Korea, also showed a difference on the degree of addiction by gender. It suggested that the female participants were more aware of their addiction based on the higher self-reporting scores [7]. At the development stage of the SAS, Kwon et al. also reported that the higher scores in the female participants were not statistically significant [9]. As for self-reporting, the female participants have the tendency to be aware and expressed their problems more openly than the male participants. According to the gender differences in adolescent symptomatology in the previous study, male students have the tendency to externalize their addiction symptoms while female participants relatively internalized them; therefore, the self-awareness showed difference by gender [18].

In this study, the SAS-SV score was 24 in boys and 28 in girls, which showed significant difference by gender. As a result, we suggested a different cut-off value by gender group through the ROC analysis reflecting the different characteristics of gender. The cut-off value, which was based on 31 boys, had a positive predictive value of 62% and a negative predictive value of 97%. Meanwhile, the cut-off value, which was based on 33 girls, had a positive predictive value of 74% and a negative predictive value of 96%. By using this figure with a negative predictive value, which is relatively higher, the predicted addiction subjects can be efficiently identified in order to prevent the addiction beforehand. In the smartphone addiction group in this study, 16.6% were boys and 26.6%, girls, which were relatively higher than those in the high-risk addiction group. These results were considered because the Smartphone Addiction Scale (SAS) is not designed to pathologically diagnose smartphone addiction but more to identify the level of the smartphone addiction risk and to distinguish the high-risk group. Smartphones are popular media that are easier to access than other media. As such, using this smartphone addiction scale as a screening tool can help prevent smartphone addiction in communities or schools.

This study was conducted in order to develop and validate the short version of the smartphone addiction scale and suggest a cut-off value for diagnosis to efficiently evaluate the smartphone addiction in the community and research areas. A total of 540 participants were included for analysis and 10 final questions were selected based on the validity determined by the experts. Both the validity and efficiency were evaluated in the 10 questions through internal consistency reliability, concurrent validity, and ROC analysis. The final questions of SAS-SV are shown in Appendix S1

The 33 questions that were previously used for the SAS were inefficient for the adolescent group due to its large number of questions and its use was limited to diagnose addiction as cut-off values were not suggested. With regards to the SAPS, which consisted of 15 questions, its cut-off value was determined through a statistical method and not through consultation with experts in its selection process with the participants. Moreover, the value was not suggested for both genders regardless of the gender differences.

As such, for this scale, questions were formed based on smartphone use, which are not found in the conventional Internet addiction scales. This scale is a short version that contains only 10 questions for easy smartphone addiction screening of adolescents who are considered vulnerable to addiction. This scale also provides a cut-off value to evaluate the level of addiction, to evaluate the treatment effect and to provide evidence of interventions different from those in the conventional scales. This scale has a high value as a screening tool because gender differences can be reflected in the results by providing a cut-off value for both genders, and the screening process that includes the evaluation by clinical psychologists is not merely the simple percentage calculation method but reflects the characteristics of the participants.

The limitation of this study revealed that the study was conducted in a particular region and the demographic factor was not fully controlled because the ratio of the gender was not 1∶1. As a result, this sample group was hard to be generalized and further studies with various sample groups should be conducted in order to evaluate the validity of this scale. Another limitation of this study concerns the use of its findings in clinical practice for diagnosis, as it was not performed under clinical settings. Therefore, further studies with well-controlled clinical settings and various participants are suggested.

However, this short-version scale was considered to be an effective means to predict the smartphone addiction based on the experts' diagnoses despite those aforementioned limitations. In addition, the SAS-SV can be used to identify a potential high-risk group for smartphone addiction, both in the community and educational fields. Further investigation of their characteristics in the future, development of program, and arrangement of plans should be taken into consideration for the prevention of smartphone addiction.

## Supporting Information

Appendix S1 **English version of SAS-SV.**
(XLSX)
